# Optogenetically evoked gamma oscillations are disturbed by cocaine administration

**DOI:** 10.3389/fncel.2013.00213

**Published:** 2013-11-27

**Authors:** Jonathan E. Dilgen, Tamas Tompa, Shalini Saggu, Thomas Naselaris, Antonieta Lavin

**Affiliations:** ^1^Department of Neuroscience, Medical University of South CarolinaCharleston, SC, USA; ^2^Faculty of Healthcare, Department of Preventive Medicine, University of MiskolcMiskolc, Hungary; ^3^Faculty of Sciences, Department of Biology, University of TabukTabuk, Saudi Arabia

**Keywords:** cocaine, gamma oscillations, prefrontal cortex, dopamine, optogenetics, *in vivo*

## Abstract

Drugs of abuse have enormous societal impact by degrading the cognitive abilities, emotional state and social behavior of addicted individuals. Among other events involved in the addiction cycle, the study of a single exposure to cocaine, and the contribution of the effects of that event to the continuous and further use of drugs of abuse are fundamental. Gamma oscillations are thought to be important neural correlates of cognitive processing in the prefrontal cortex (PFC) which include decision making, set shifting and working memory. It follows that cocaine exposure might modulate gamma oscillations, which could result in reduced cognitive ability. Parvalbumin-positive fast-spiking interneurons play an orchestrating role in gamma oscillation induction and it has been shown recently that gamma oscillations can be induced in an anesthetized animal using optogenetic techniques. We use a knock-in mouse model together with optogenetics and *in vivo* electrophysiology to study the effects of acute cocaine on PFC gamma oscillation as a step toward understanding the cortical changes that may underlie continuous use of stimulants. Our results show that acute cocaine administration increases entrainment of the gamma oscillation to the optogentically induced driving frequency. Our results also suggest that this modulation of gamma oscillations is driven trough activation of D1 receptors. The acute cocaine-mediated changes in mPFC may underlie the enhancement of attention and awareness commonly reported by cocaine users and may contribute to the further use and abuse of psychostimulants.

## INTRODUCTION

Use of psychostimulants such as cocaine is a serious health problem and opens the door to neurobiological changes in limbic and cortical circuits that engage cognitive and emotive processing. Recently, we have just began to understand the cellular adaptations that occur in cortex following a single exposure to cocaine and their contribution to the continuous and further use of drugs of abuse.

The behavioral consequences of first time cocaine use are varied and appear to be somewhat contradictory. First time cocaine users often report feeling a sharpening of the senses (Ashley and Hitzemann, 1990), and anecdotal information suggest that acute cocaine increases attention. Indeed, individuals with ADHD will sometimes self-medicate with cocaine (Weiss and Mirin, 1986). Contrastingly, Jentsch et al. (2002) have shown that acute cocaine administration impairs performance on a reversal learning task, and several studies have reported compromised performance during repeated acquisition tasks in monkeys (Thompson and Moerschbaecher, 1979; Evans and Wenger, 1992). Additionally, imaging studies in humans have shown that acute cocaine administration induces prominent activation of the prefrontal cortex, primarily in the dorsolateral regions (Howell et al., 2010). Furthermore, acute cocaine administration has been linked to poor impulse control (Fillmore et al., 2002; Jentsch et al., 2002; Garavan et al., 2008). Therefore, it seems that first time cocaine use may give users a sense of enhanced awareness, while cognitive performance is diminished.

Neuronal oscillations are thought to be general and fundamental mechanisms for enabling coordinated activity during normal brain functions (Engel and Singer, 2001; Buzsáki and Draguhn, 2004; Jutras et al., 2009) and it is within this framework that the function of gamma oscillations have recently acquired importance. Gamma oscillations in the cortex involve the reciprocal interaction between interneurons, mainly PV+ fast spiking interneurons (FS PV+) and principal cells (Traub et al., 1997). The predominant mechanism underlying this type of rhythm is the phasic excitation (via fast, AMPA receptor mediated PSPs) of interneurons following orthodromic spike generation in principal cells (Parra et al., 1998). At spike rates in the tens of Hz, the massive convergence of local excitatory inputs onto interneurons is sufficient to overcome frequency-dependent and metabotropic receptor-mediated (Parra et al., 1998; Vignes et al., 1998) attenuation of postsynaptic responses, and is therefore able to produce large (2–10 mV at rmp) compound EPSPs. The resulting divergence of outputs from individual interneurons back to the principal cell leads to temporally modulated principal cell output, provided, of course, that different interneurons fire synchronously.

Gamma oscillations appear to be a critical mechanism underlying the cognitive and behavioral function of mPFC. It is therefore highly likely that gamma oscillations in mPFC would be altered by cocaine administration. In this study we investigated changes in gamma oscillations following an acute administration of cocaine. Our studies show that acute cocaine administration narrows the bandwidth of gamma oscillation responses evoked by optogenetic stimulation, resulting in precise entrainment at 40 Hz. Furthermore, pharmacological studies suggest that the cocaine effect is mediated by DAergic receptors.

Since PV+ interneurons are an integral part of the generation of gamma oscillations (Traub et al., 1997), the data presented here also support the hypothesis that acute cocaine administration affects gamma oscillations via DAergic modulation of fast spiking, PV+ interneurons.

## MATERIALS AND METHODS

All procedures were done in accordance to the National Institute of Health guidelines as approved by the Medical University of South Carolina Institutional Animal Care and Use Committee.

### AAV INJECTION

Male PV-Cre mice (B6; 129P2-Pvalb^tm1(Cre)Arbr/J^ Jackson Laboratory (Bar Harbor, ME, USA) were anesthetized with ketamine/xylazine (120 mg/kg ketamine, 15 mg/kg xylazine) and secured in a mouse/neonate rat adapter (51625, Stoelting, Wood Dale, IL) fit to a stereotaxic apparatus (Narashige, Japan). Using aseptic technique, a small burr hole was drilled over the medial PFC, (2.0 mm anterior to Bregma, 0.5 mm lateral from the midline) of each hemisphere with a dental drill. Animal core temperature was maintained at 37.5 ± 0.5°C using a heating pad. The viral vector (AAV2/5.EF1a.DIO.hChR2(H134R)-EYFP.WPRE.hGH, Penn Vector Core, University of Pennsylvania; 5–50 × 10^6^ particles per μl in 10% sucrose 0.1 M PBS) was delivered via a glass micropipette driven by a microinjector (Nanoject II, Drummond Scientific Company, Broomall, PA, USA). After being loaded with virus suspension, the injection pipette was lowered into the medial PFC (-1.0 mm ventral from the pial surface) and five injections of 41.4 nl each (207 nl total) were given at 1 min intervals (rate = 23 nl/s). The pipette was left in place for 3–5 min before being withdrawn from the brain. The injection procedure was then repeated on the contralateral side. The burr holes were then covered with bonewax, the skin was repositioned and closed with sutures. Animals were returned to their home cages after regaining movement and a post-injection analgesic was given.

### IMMUNOHISTOCHEMISTRY

To confirm the selective expression of YFP in PV-containing neurons, mice were deeply anesthetized with ketamine/xylazine and transcardially perfused with paraformaldehyde (4%, Sigma). After 24–48 h in paraformaldehyde, brains were transferred to a 30% sucrose solution overnight. Coronal (40 μm) sections were cut using a cryostat and alternating sections were collected for subsequent immunohistochemistry.

Dual-labeling to visualize the ChR2-YFP conjugate and PV was achieved by first incubating sections in a blocking solution consisting of 0.3% Triton, 1% BSA (OmniPur, EMD4biosciences), 5% normal goat serum (Abcam), in PBS for 1 h. Sections were then incubated for 24 h at 4°C in rabbit-anti-PV(ABCAM) diluted at 1:1,000 in the same blocking solution. After 24 h, sections were thoroughly rinsed several times in PBS followed by incubation in a goat antirabbit IgG secondary antibody conjugated to the Alexa 594 fluorophore (Invitrogen) used at 10 μg/ml in 1% BSA in PBS for 1–2 h in the dark on a shaker, washed and mounted with Prolonged Gold antifade reagent (Invitrogen).

Sections were examined with a confocal laser-scanning microscope (Leica TCS Sp5) using a Plan Apochromatic 63× objective. Images were acquired using sequential acquisition of the different channels to avoid cross-talk between fluorophores.

### ELECTROPHYSIOLOGICAL RECORDINGS

Three to six weeks following viral infection, animals were anesthetized as described above (initiation by Ketamine/xylazine, 0.5–1% isoflurane – O_2_/CO_2_ anesthesia was administered through a gas anesthesia platform for mice (50264, Stoelting, Wood Dale, IL, USA), and adjusted to maintain adequate anesthesia levels (monitored by toe pinch reflex and breathing rate), positioned in the stereotaxic apparatus with mouse adapter. Animal core temperature was maintained at 37.5 ± 0.5°C using a heating pad and DC Temperature Controller coupled to a thermal probe (40-90-8, FHC, Bowdoin, ME, USA). A midline incision was made over the recording site, the scalp retracted, and a small craniotomy was drilled around the injection site. Remaining bone was removed and dura mater was resected. A custom made optrode consisting of a 0.5 mm borosilcate glass pipette (7–10 M Ohms) glued to a 50 μm diameter optic fiber (Thorlabs, Inc, Newton, NJ, USA) was constructed. The recording pipette was filled with 2 M NaCl and 2% Chicago Sky Blue was added to prevent light-induced artifact. As an extra precaution, care was taken to ensure the tip of the recording pipette was not directly in the laser beam emitting from the optic fiber: approximately 200 μm of space was left from the tip of the optic fiber to the tip of the recording pipette. The optrode was lowered into the cortex and 30 min was allowed to elapse to allow the brain tissue to “settle” around the optrode. The optrode was lowered through the brain using a Narishige (Japan) hydraulic microdrive. Extracellular signals were amplified by a Grass amplifier (Grass Technologies, West Warwick, RI, USA), digitized at 10 kHz by a 1401plus data acquisition system, visualized using Spike2 software (Cambridge Electronic Design, LTD., Cambridge, UK) and stored on a PC for offline analysis. Line noise was eliminated by using a HumBug 50/60 Hz Noise Eliminator (Quest Scientific Inc., Canada). The signal was band-pass filtered online between 0.1 and 10 kHz for single- or multi-unit activity, or between 0.1 and 130 Hz for local field potentials (LFP) recordings.

Light stimulation was generated by a 473 nm laser (DPSS Laser System, OEM Laser Systems Inc, East Lansing, MI, USA), controlled via a 1401plus digitizer and Spike2 software (Cambridge Electronic Design LTD., Cambridge, UK). Light pulses were delivered via the 50-μm diameter optical fiber glued to the recording electrode (Thorlabs, Inc, Newton, NJ, USA). After tissue stabilization, single unit recordings were obtained and the minimal amount of laser power needed to elicit a response was found. After establishing this parameter, laser power was subsequently fixed at minimum amount of laser power +25% more.

Extracellular recordings were made from single neurons and multiunits in the prelimbic and infralimbic part of the mPFC with low impedance glass pipette electrodes, using the following stereotaxic coordinates: 2 mm anterior to bregma; 0.5 mm lateral to midline; 1 mm vertical Recorded signals were amplified, band-pass filtered according to the needs (l fp: 1–200 Hz, units: 300 Hz–3 kHz), displayed on analog oscilloscope, digitized (1401 plus interface, Cambridge Electronic Design), and recorded on a Pentium 4 personal computer (PC) using Spike2 software (version 4; Cambridge Electronic Design).

Individual neurons were identified by the configuration, shape, and amplitude of the action potentials (spikes). Pyramidal neurons can be distinguished from interneurons based on their broader action potential waveform (peak-to-valley >500 μs) and lower baseline discharge rate (<10 Hz). The broader waveform means wider half-width, which is measured at 50% of height of the AP. Spikes in the spike2 system are detected and recorded based on the waveform signal that crosses a trigger level and matches a pre-set shape or template, which is created for the individual neuron at the beginning of the recording period.

### EXPERIMENTAL PROTOCOL

At the top of the recording track the efficacy of optical stimulation was assessed by monitoring single-unit or multi-unit responses to various light pulses (duration 10–250 ms). High firing rate action potentials, low half-width amplitude (presumably from PV-positive interneurons) during the light stimulation, and/or the inhibition of regular spiking units was considered confirmation of optical stimulation of ChR2 expressing PV+ interneurons. The optrode was repositioned along the dorsal ventral axis if no response was found. Upon finding a stable response, filters were changed to record field potentials (0.1–100 Hz). Optical stimulation was delivered for 3 s at 40 Hz (120 pulses, 1 ms duration). Field potential activity was monitored for a minimum of 10 min while occasionally stimulating at 40 Hz to ensure the stability of the electrode placement and the ability to induce the oscillation. Additionally LFP activity was monitored as a tertiary method of assessing anesthesia levels. Several animals were excluded from analysis due to fluctuating levels of LFP activity that resulted from titration of anesthesia levels during the experiment. Optical stimulation was repeated immediately before and after the administration of cocaine (15 mg/kg, i.p.) and then repeated every 2 min for a total of 30 min. This was immediately followed by injection of a selective dopamine receptor antagonist (D1 antagonist SCH23390, 1.0 mg/kg, i.p. or the D2 antagonist Sulpiride, 15 mg/kg, i.p.) and optical stimulation. Again, light stimulation was repeated every 5 min for 30 min. Additionally, a several points throughout the recording a 3 s long 8 Hz train stimulation (1 ms pulses, ISI = 124 ms) was administered as a control for specificity. It has been demonstrated that 8 Hz stimulation does not enhance 8 Hz oscillations in animals which have ChR2 expressed in PV+ interneurons, but 8 Hz stimulation will enhance 8 Hz oscillations in animals where ChR2 is expressed in pyramidal neurons (16). In several other animals, a shorter 40 Hz stimulation was used which consisted of 10 light pulses, each 10 ms in duration, with 15 ms between pulses. These recordings were included in the assessment of changes in peak power.

### DRUGS

Drugs were delivered through a secure i.p. line implanted in aseptic conditions before placing the animal in the stereotaxic frame. Drugs were dissolved in physiological saline or DMSO (sulpiride) and administered intraperitoneally sulpiride, 15 mg/kg (17–19); SCH23390 [R(_)-7-chloro-8-hydroxy-3-methyl-1-phenyl-2,3,4,5-tetrahydro-1H-3-benzazepine hydrochloride], 1.0 mg/kg (20–22). Cocaine HCl, SCH 23390, sulpiride, and other reagents were purchased from Sigma (St. Louis, MO, USA). Infusions of physiological saline were used as a control but not DMSO.

### ANALYSIS AND STATISTICS

Local field potentials signals were analyzed using a custom Matlab (MathWorks) script. The power of the induced oscillation was assessed by comparing the power spectral densities in the 3 s prior to stimulation, to that in the 3 s during the stimulation. Relative power and the power ratio were assessed using methods similar to Cardin et al. (2009). Briefly, relative power was calculated by dividing the power in a band (varying from 0 to 20 Hz; see **Figure 5**) centered on the stimulation frequency (i.e., 40 Hz), by the total power present in the power spectrum from 0 to 100 Hz. The power ratio is defined as the relative power while the stimulation is on divided by the relative power while the stimulation is off.

The effectiveness of inducing oscillations with optical stimulation was assessed by comparing the relative power in an 8 Hz band before and during the stimulation (see **Figures 3C,D**). Drug effects were assessed by comparing relative power as well as peak power (power at 40 Hz) among drug conditions. Relative power assessments consisted of only those recordings in which 3 s long optical stimulation was utilized, while peak power assessments also included recordings with a different stimulation pattern (i.e., 25, 10 ms duration light pulses at 40 Hz). Differences in power were assessed using the Student’s *t*-test or one-way ANOVA, unless otherwise noted.

To measure the effects of cocaine administration on gamma oscillations, power ratios were calculated for each recording under naïve and cocaine conditions. For each recording, power-ratio decay curves were calculated by varying between 0 and 20 Hz the size of the frequency band used to calculate the power ratio (in all cases the band was centered at 40 Hz). Power-ratios appeared to decay as a hyperbolic function of the size of the frequency band:

$$r\,\Delta =r_{0}/\left(1+\alpha \,\Delta \right)$$

where *r*Δ is the power-ratio for a given frequency band of size Δ (centered at 40 Hz), the scale factor *r*_0_ is the power-ratio ratio for a band of size 0, and the decay factor α is the rate at which the power-ratio decays with increasing frequency band size. For large values of α the power-ratio will decay rapidly as the frequency band size increases. Therefore, large alpha values indicate a gamma oscillation that is tightly entrained to the stimulation frequency (40 Hz). The parameters *r*_0_ and α were estimated by fitting a hyperbolic function to the empirically measured power-ratio decay curves using the *lsqnonlin* utility in MATLAB. Fits were made for each recording independently for a total of *n* = 12 fits (6 subjects × 1 observation × 2 conditions).

We used a permutation test to determine if cocaine administration induced significant changes in *r*_0_ and/or α. The *r*_0_ and α parameters were re-estimated for all possible permutations of the naive and cocaine conditions. This resulted in a null distribution of median (across subjects) differences in the *r*_0_ and α values between the naive and cocaine conditions. The statistical significance of the median *observed differences* in *r*_0_ and α were determined with respect to this null distribution.

## RESULTS

### CELL TYPE SPECIFIC EXPRESSION OF ChR2

**FIGURE 1:**
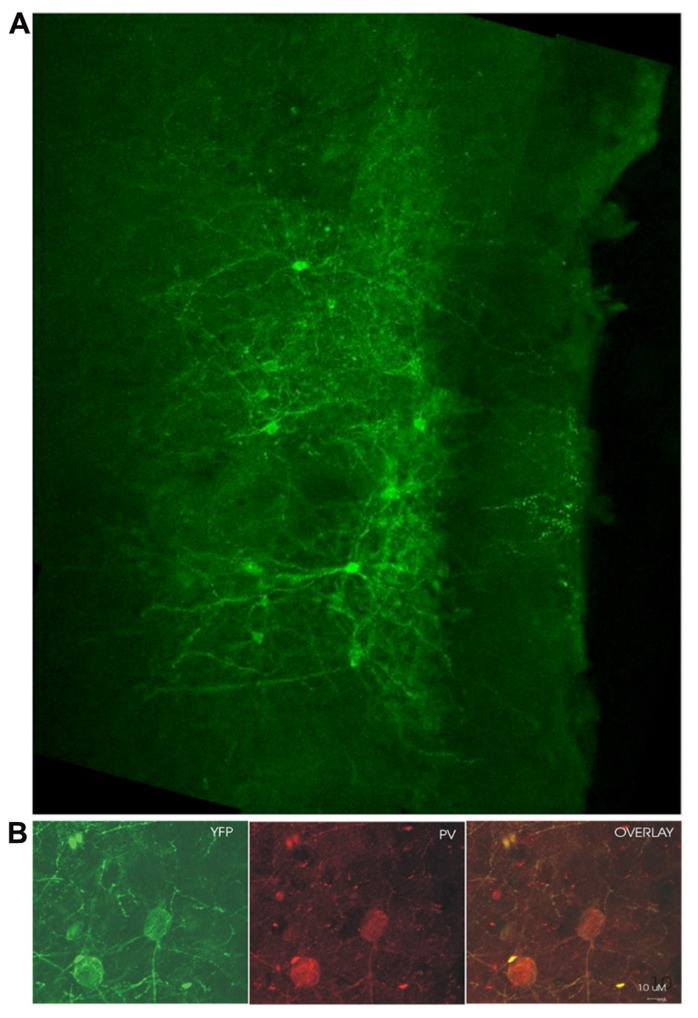
**ChR2 expression in PV+ cells 4 weeks post-infection.** **(A)** YFP fluorescence in a medial PFC coronal section from an infected mouse (medial/pial surface is to the right, dorsal toward the top of the page)*.* **(B)** YFP labeled cells double-stained for parvalbumin (PV). YFP labeling in green on the left, PV immunoreactivity shown in red in the middle, overlay of YFP and PV labeling on the right. Yellow suggests colabelling of PV+ cells with YFP, demonstrating ChR2 expression in cells which are immunoreactive for PV.

**FIGURE 2:**
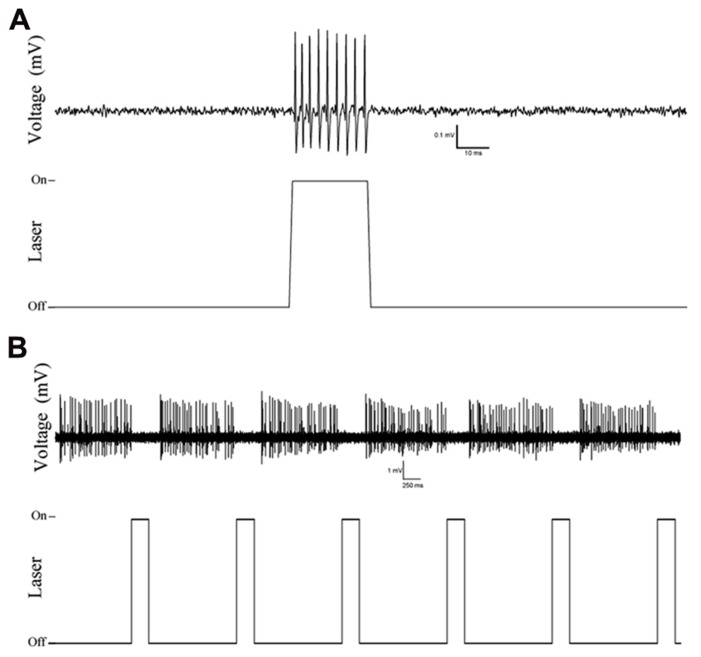
**Example traces of single and multi-unit recordings in Cre-PV+ mice 4 weeks after injection of AAV-ChR2-YFP construct showing selective responses to laser stimulation.** **(A)** Single unit action potentials are evoked by laser stimulation, illustrating the isolation of a putative PV+ interneuron, and activation of putative PV+ fast-spiking interneurons*.* **(B)** Multi-unit recording showing inhibition of regular firing units during optical stimulation. **(A,B)**: top traces are extracellularly recorded potentials (band-pass filtered: 30–300 Hz), bottom traces indicates the timing of optical stimulation.

Examination of coronal PFC sections using confocal microscopy confirmed successful infection of the medial PFC via expression of the reporter protein YFP (**Figure 1A**). This staining was confirmed to be in PV+ interneurons through the double-labeling of PV and YFP (*n* = 55 cells, 49 cells labeled for YFP, 53 cells labeled for PV, 47 cells double labeled, 10 sections, **Figure 1B**). The infectiosn encompassed the cingulate cortex and prelimbic cortex, in some cases, also the dorsal infralimbic cortex was infected. To confirm light activation of the ChR2 protein, single, multi-unit (MU) and LFP recordings were performed in anesthetized mice 4–6 weeks after injection of the virus into the medial PFC. Single-unit recordings confirmed that putative FS PV+ neurons were excited by laser stimulation (**Figure 2A**). MU recordings provide evidence that laser activation at various durations elicited an inhibition of MU activity during the stimulation (**Figure 2B**). This reflects a pause in spontaneous firing among principal neurons as a consequence of the inhibition presumably caused by light-evoked excitation of PV+ GABAergic interneurons.

### ACTIVATION OF FS PV+ INTERNEURONS GENERATES GAMMA OSCILLATIONS: EFFECTS OF ACUTE COCAINE ADMINISTRATION

**FIGURE 3:**
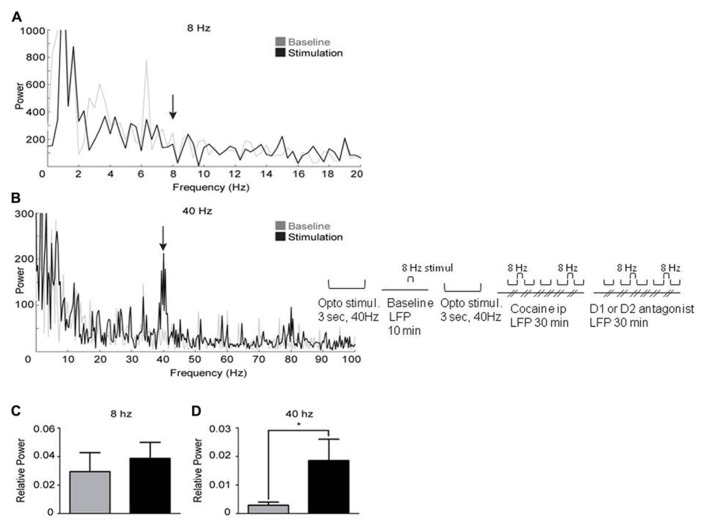
**Activation of PV+ interneurons in the medial prefrontal cortex generates gamma oscillations.** Power spectral densities from 3 s epochs occurring just prior to (gray) or during (black) optical stimulation delivered at 8 Hz **(A)** or 40 Hz **(B)**. **(A)** 8 Hz stimulation was ineffective at inducing oscillations. **(B)** Stimulation at 40 Hz induces a clear peak in the gamma range (37–43 Hz). **(C**, **D)** represent the relative power induced by laser stimulation (see Methods). **(C)** Optical stimulation at 8 Hz did not significantly increase oscillations. **(D)** In contrast, optical stimulation at 40 Hz significantly increased oscillations, demonstrating successful induction of gamma oscillations (paired, one-tailed, Student’s *t*-test: *p* < 0.05, *n* = 6).

**FIGURE 4:**
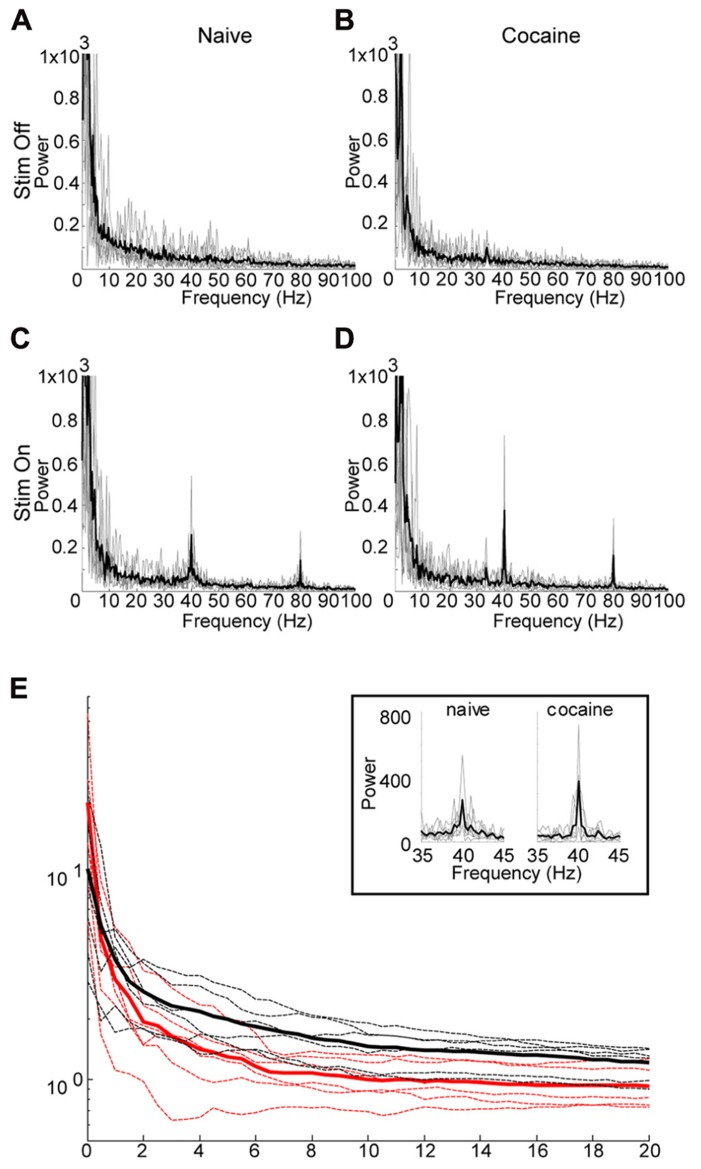
**Effect of Cocaine on the induced oscillation.** **(A)** through **(D)** are power spectra (0–100 Hz) for six subjects which received 40 Hz optical stimulation for 3 s duration. Thin gray lines represent individual data, while the thicker black line represents the mean of the individual data. In a few cases significant line noise was present at precisely 60 Hz, this was excluded from the graphs by setting the power at 60 Hz to the mean of the power at 59 and 61 Hz. **(A**, **C)** are prior to i.p. cocaine injection while **(B**,**D)** are after. **(A**,**B)** represent immediately preceding the optical stimulation, while **(C**,**D)** are during stimulation. In **(C**,**D)**, there is a clearly distinguishable peak at the stimulation frequency, with a harmonic echo at 80 Hz. The shape of the peak of the induced oscillation in the cocaine treated animals **(D)** appears to be sharper, with increased peak power relative to the naïve condition **(C)**. **(E)** Comparison of power ratios across bandwidth frequencies from 0 to 20 Hz. Power ratio values plotted against the bandwidth “window” surrounding the 40 Hz stimulation frequency. Cocaine appears to have a sharpening effect on the induced oscillation reflected here as an increase in peak power, while decreasing the bandwidth of the induced oscillation. However, only the change in bandwidth is significant (*p* = 0.008, see text). Power ratio values are shown for cocaine (red) and naïve (black) conditions. Thicker solid lines represent means, dashed lines are individual animals. The boxed inset depicts an expanded view of the induced oscillation at 40 Hz under naïve and cocaine conditions.

It has been demonstrated that synchronous activation of FS interneurons from 8 to 200 Hz generates gamma oscillations (Cardin et al., 2009). Therefore, activating FS interneurons in the mPFC of Cre-PV mice infected with floxed ChR2-YFP at gamma frequency is expected to elicit a gamma-elevation in the LFP. FS interneurons were activated in 14 infected mice at 8 and 40 Hz with light pulses (1 ms duration) while recording LFP’s. Laser stimulation at 40 Hz elicited a robust increase in gamma power near 40 Hz (**Figures 3B,D** and **4A,C**). However, light stimulation at 8 Hz did not produce an increase in power near 8 Hz (**Figures 3A,C**). These findings confirm previous work by Cardin et al. (2009), demonstrating that a cortical network containing ChR2 expressing PV+ interneurons will produce light-induced oscillations in the gamma range, but not at lower frequencies. This result also suggests that the evoked 40 Hz oscillation observed in the LFP is not a product of light-induced artifacts. Furthermore, stimulation at 40 Hz produces an increase in relative power in the gamma range (**Figure 3D**). Taken together, these findings suggest that light-induced gamma oscillations in this preparation are indeed the product of a physiological network effect.

Following successful optical induction of gamma oscillations, we investigated the effect of acute systemic cocaine administration (15 mg/kg, i.p.) upon this phenomenon. Due to fluctuating anesthesia levels, resulting in unstable background activity, several animals were excluded from the analysis (see Methods). Cocaine administration appeared to have maximal effect at 15 min post-injection. Following cocaine administration, the induced oscillation appeared to have increased peak power, with a simultaneous decrease in bandwidth (**Figures 4C,D**). However, further investigation revealed that the peak power is not significantly altered (median difference in *r*_0_ between cocaine and naive = 8.0022, *p* = 0.11, permutation test). Contrastingly, the bandwidth of the induced oscillation was significantly decreased after cocaine as evidenced by a significant increase in the decay of the power-ratio with increasing band size (median difference in alpha between cocaine and naive = 2.3370, *p* < 0.01, permutation test). Therefore, the main effect of cocaine is that it increases the entrainment of the laser-induced oscillation to the driving frequency, resulting in a very narrow-bandwidth gamma oscillation centered at 40 Hz.

### DOPAMINE MODULATES COCAINE EFFECTS ON GAMMA OSCILLATIONS

**FIGURE 5:**
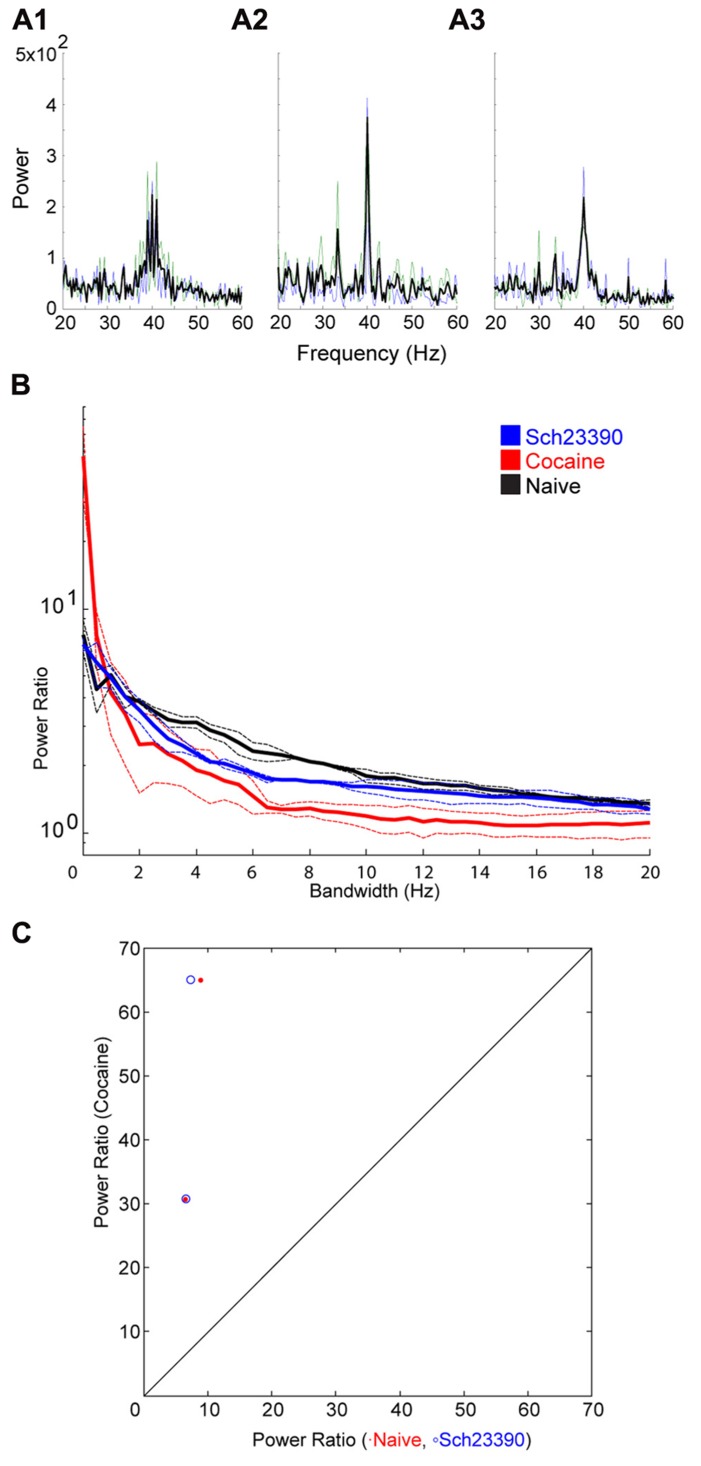
**Effect of the D1 antagonist SCH23390 on the cocaine induced change to the induced oscillation.** **(A)** Power spectra around the stimulated frequency in naïve (A1), cocaine (A2), and SCH23390 (A3) conditions. Sharpening of the peak is seen here again and appears to be reversed by SCH23390. **(B)** Power ratio vs. bandwidth plot reflecting the changes in peak power and bandwidth seen above. SCH23390 appears to block the sharpening effect of cocaine treatment. **(C)** Comparison of power ratio at the peak, across treatment conditions. Above the line represents a larger power ratio in cocaine treatment vs. either naïve (red) or SCH23390 (blue). Data points are nearly overlapping demonstrating that SCH23390 appears similar to the naïve condition.

**FIGURE 6:**
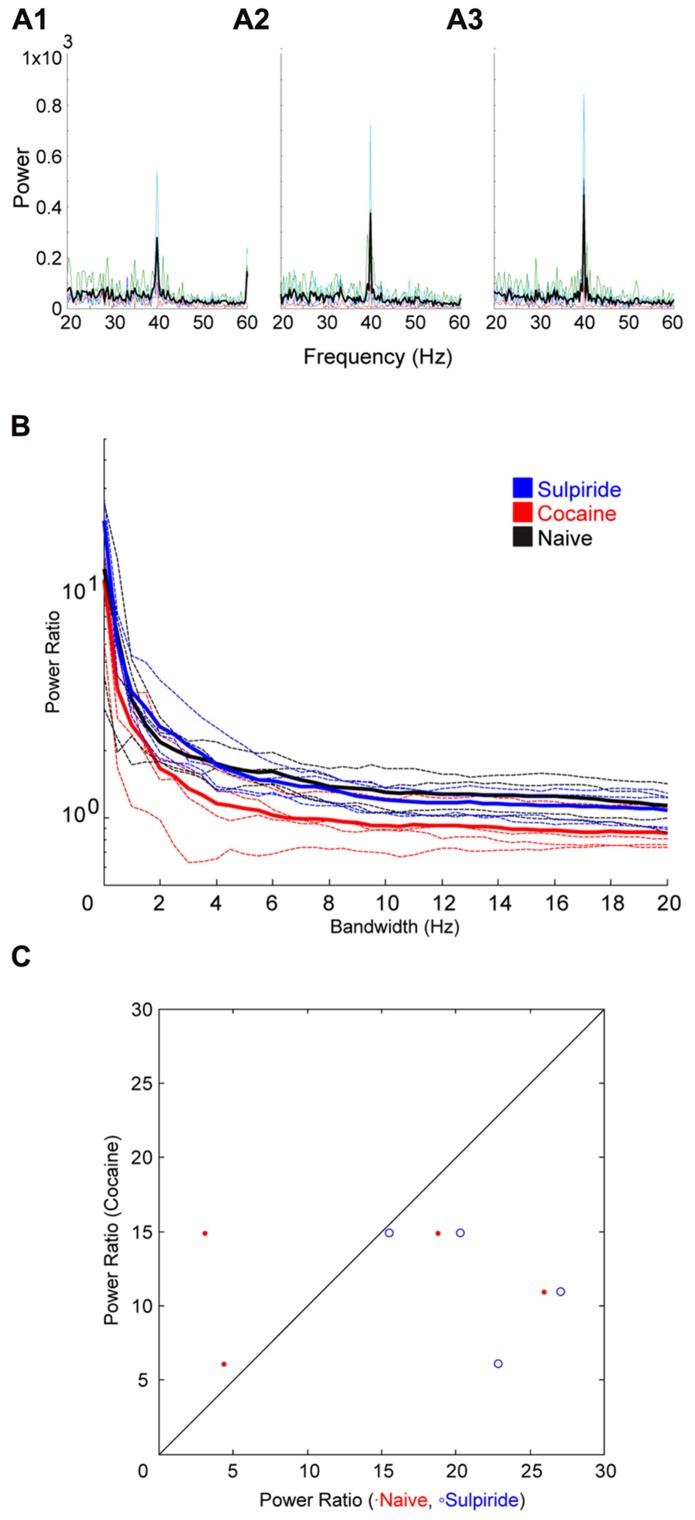
**Effect of the D2 antagonist Sulpiride on cocaine induced change to the induced oscillation.** **(A)** Power spectra around the stimulated frequency in naïve (A1), cocaine (A2), and Sulpiride (A3) conditions. Sharpening of the peak is seen here again and appears to be enhanced by Sulpiride. **(B)** Power ratio vs. bandwidth plot reflecting the changes in peak power and bandwidth seen above. Sulpiride appears to increase the sharpening effect of cocaine treatment. **(C)** Comparison of power ratio at the peak, across treatment conditions. Above the line represents a larger power ratio in cocaine treatment vs. either naïve (red) or Sulpiride (blue). Sulpiride data points are shifted to the right demonstrating that Sulpiride may increase the peak power further than cocaine.

Following cocaine injections, either a D1 or D2 selective antagonist was systemically administered. The D1 antagonist SCH23390 significantly blocked the effect of cocaine on bandwidth (**Figure 5**). The permutation test revealed that both the scale factor and the decay factor of the hyperbolic function fitted to the data were significantly different in SCH23390 vs. cocaine conditions (*p* < 0.01, *n* = 2, permutation test). Conversely, the D2 antagonist sulpiride appeared to enhance the effect of cocaine (**Figure 6**). However, this effect was not significantly different in sulpiride vs. cocaine conditions (*p* > 0.05, *n* = 4, permutation test). These results suggest that perhaps the effects of cocaine are being mediated through activation of D1 receptors; however, the “*n*” of these experiments is small.

In a different set of experiments we investigate the contributions of D1and D2 receptors on laser-evoked gamma oscillations. We systemically administered either SCH23390 or sulpiride and compared the laser-evoked oscillations before and after administration of the drug. The D1 antagonist SCH23390 had a trend toward decreasing the peak of the evoked response (*p* = 0.066, MATLAB permutation test, *n* = 5 animals), while the bandwidth had a trend towards being more narrow (*p* = 0.138, MATLAB permutation test). On the other hand, the DAD2 antagonist sulpiride did not have an effect on either the peak or the bandwidth of the evoked response (*p* = 0.237 and 0.254 respectively, MATLAB permutation test, *n* = 3 animals). Thus, it appears that D1 receptors might contribute to some characteristics of laser-evoked gamma oscillations, suggesting a role for dopaminergic regulation of these oscillations, and by extension of cognitive processes which are believed to rely upon such oscillations. Moreover, these data support the suggestion that changes in laser-evoked gamma oscillations are mediated through activation of D1 receptors.

In summary, we have successfully demonstrated the expression of ChR2 in PV+ interneurons as exhibited via immunohistochemistry and electrophysiology. Additionally, we have confirmed that laser activation of PV+ interneurons is sufficient to induce gamma oscillations *in vivo*, as originally shown by Cardin et al. (2009) and Carlén et al. (2012). Furthermore, we have demonstrated that acute cocaine administration can reduce the bandwidth of the optically induced oscillations and activation of D1 receptors affects gamma oscillations. These findings suggest that cocaine may not affect the power of induced oscillation, but rather changes its character as evidenced by the sharpening of the bandwidth. This result may reflect an increase in synchronicity during the induced oscillations.

## DISCUSSION

Our results show that acute cocaine administration alters the nature of laser-evoked gamma oscillations.

Gamma oscillations in cortex, including the PFC, involve the reciprocal interaction between fast spiking, PV+ interneurons and principal cells (Traub et al., 1997). Given our previous results demonstrating that cocaine administration affects the activity of FS PV+ inteneurons (Kroener and Lavin, 2010), we hypothesized that cocaine administration would alter laser-evoked gamma oscillations in the medial PFC. Our results show that indeed, acute cocaine administration increases entrainment of optically induced gamma oscillations.

Following acute cocaine administration we observed a significant decrease in the spread of the induced oscillation. This suggests that the firing of principal neurons reflected in the oscillation becomes more synchronous following cocaine administration (i.e., increased entrainment). An increase in synchronicity suggests increased entrainment of the cortical network leading to alterations in cortical processing. We suggest that this could be the mechanism by which cocaine increases attention and awareness in first time users. For example, first time cocaine users often report feeling a sharpening of the senses (Ashley and Hitzemann, 1990). The misapprehended introspective impression could reflect the tightening of prefrontal processing systems. This short-live feeling of enhanced awareness and attention mediate by increases in gamma oscillations may motive the user to keep using and increasing the doses of stimulants in an effort to keep the feelings of euphoria constant.

Moreover, our results show that activation of D1R seems to mediate the increase in gamma synchronicity elicited by acute cocaine.

While dopamine plays a well documented role in attention and working memory (for review see: Robbins and Arnsten, 2009) until very recently there was a dearth of information regarding the role of DA in mediating oscillatory activity related with these executive functions. Several authors reported that systemic administration of amphetamine, apomorphine, or metamphetamine do not affect gamma oscillations in rat cortex or hippocampus (Ma and Leung, 2000; Pinault, 2008; Ehrlichman et al., 2009), but recently in a very elegant review, Furth et al. (2013) suggest that D4R in PFC and hippocampus may play a role in regulating gamma oscillations, and Dzirasa et al. (2006, 2009) have shown that DAT1knockout mice have increased hippocampal gamma power and enhanced hippocampus-PFC synchronicity, whereas Demiralp et al. (2007) using EEG data, have shown that DA increases the power of cortical oscillations.

Studies *in vitro* have shown contradictory results regarding the role of DA in gamma oscillations: whereas pharmacological induction of gamma oscillations in PFC was reduced by bath application of haloperidol (Jones et al., 2012), in CA1 DA increased the power and duration of electrical-induced gamma oscillations (Wójtowicz et al., 2009). On the other hand, when oscillations in CA1 were chemically evoked, haloperidol decreased the power and duration (Weiss et al., 2003), and application of D1R agonists decreased chemically induced gamma power (Weiss et al., 2003). These data show that the effects of DA may depend on the different means used to elicit gamma oscillations, as chemically induced gamma and electrically- induced gamma are underlined by different mechanisms.

Whereas the effects of DA in gamma oscillations are not yet clear, we have previously demonstrated that DA has a biphasic effect on sIPSCs. At lower doses, DA increased sIPSCs amplitude via activation of D1R, whereas at higher doses DA decreases sIPSCs amplitude via activation of D2R (Trantham-Davidson et al., 2004; Kroener and Lavin, 2010).

Furthermore, we have demonstrated that activation of DAD1 receptors increases intrinsic excitability of pyramidal cells (Lavin and Grace, 2001). Thus, we propose that acute cocaine administration is increasing endogenous DA to levels that activate both D1 and D2 receptors eliciting a decrease in amplitude of sIPSCs (noise), and an increase in intrinsic excitability (signal), thereby, augmenting the temporal precision and increasing the entrainment of FSPV+ interneurons.

## CONCLUSION

In conclusion, we propose that acute cocaine administration may disrupt normal cognitive functioning via increase of synchronicity in gamma oscillations, thus altering cortical networks and disrupting cognitive processes.

## Conflict of Interest Statement

The authors declare that the research was conducted in the absence of any commercial or financial relationships that could be construed as a potential conflict of interest.

## AUTHOR CONTRIBUTIONS

Jonathan E. Dilgen and Tama Tompa contributed equally. They performed the experiments, analyzed the data and contributed to writing the manuscript. Shalini Saggu performed the YFP-Chr double labeling and generated the pictures. Thomas Naselaris performed the analysis. Antonieta Lavin supervised the experiments and wrote the manuscript.
